# Significance of hind wing morphology in distinguishing genera and species of cantharid beetles with a geometric morphometric analysis

**DOI:** 10.3897/zookeys.502.9191

**Published:** 2015-05-04

**Authors:** Junyan Su, Kaile Guan, Jiaxu Wang, Yuxia Yang

**Affiliations:** 1 The Key Laboratory of Zoological Systematics and Application, College of Life Sciences, Hebei University, Baoding 071002, Hebei Province, China

**Keywords:** Geometric morphometrics, hind wing morphology, Cantharidae, taxonomy

## Abstract

There remain some difficulties in delimitation of related genera or sibling species for cantharid beetles, because the traditionally taxonomic method and morphological characters have not been updated or introduced. In the present study, we firstly use the landmark-based geometric morphometrics to analyze and compare the hind wings of nine species belonging to three genera of Cantharinae to ascertain whether this approach may be used as a reliable method in the study of the taxonomy of this group. The results show that the shape differences of the hind wings among genera seem more variable than that within each genus, and the variations for each species are different from one another, as shown in the principal component analyses. And the canonical variates analyses show that there are significant differences among the genera and the species of each genus, which demonstrates that the hind wing shape can be diagnostic for both generic and specific identification of the cantharid beetles. This study sheds new light into clarifying the taxonomic uncertainties of Cantharidae, and lays a foundation for further studies on the evolution of the cantharid hind wing shape.

## Introduction

The Cantharinae represents a subfamily of beetles belonging to the family Cantharidae (Bouchard et al. 2011). To date, it has approximately 2000 species belonging to 43 genera (Yang 2010, Švihla 2011), which are widely distributed in the Holoarctic and Oriental regions (Brancucci 1980). Traditionally, the taxonomy of this group is mainly based on the structure of male genitalia and tarsal claws. However, it is impossible to accurately identify all species by only using these characters, especially for the morphologically similar sibling species, such as *Falsopodabrus himalaicus* species complex (Yang et al. in press). Moreover, it is not easy to clarify the status of some species among the related genera, such as Habronychus (Monohabronychus) multilimbatus (Pic, 1910), which was transferred several times (Okushima 2003, Švihla 2004, Brancucci 2007) in the *Stenothemus* genera complex (Švihla 2004). These difficulties underline the need for further studies to clarify the taxonomy of cantharid beetles either by searching for new morphological characters of high diagnostic value or applying alternative effective methods.

It is well-known that wing shape of insects exhibits a high heritability in nature (Bitner-Mathé and Klaczko 1999, Moraes et al. 2004), wing morphology is of a primary importance to entomologists interested in systematics. It was Comstock (1893) who first popularized the use of insect wing venation for traditional classification (Kunkel 2004). Since the 1970’s, several authors have begun to use the insect wings especially 2D morphometrical studies in systematics and phylogeny (Plowright and Stephen 1973, Rohlf 1993, Klingenberg 2003, Gumiel et al. 2003). Geometric morphometrics utilizes powerful and comprehensive statistical procedures to analyze shape differences of a morphological feature, using either homologous landmarks or outlines of the structure (Rohlf and Marcus 1993, Marcus and Corti 1996, Adam et al. 2004), and it is considered to be the most rigorous morphometric method (Gilchrist et al. 2000, Debat et al. 2003). Wings are excellent structure for studying morphological variation because they are basically 2-dimensional and the venation provides many well-defined morphological landmarks (Gumiel et al. 2003), the interactions of the veins, which are easy for identification and able to capture the general shape of the wing (Bookstein 1991). Among insects, the use of geometric morphometric analysis to study wing venation has been useful in identification at the individual level (Baylac et al. 2003, Dujardin et al. 2003, Sadeghi et al. 2009), in distinguishing sibling species (Matias et al. 2001, De la Riva et al. 2001, Villegas et al. 2002, Klingenberg and Savriama 2002, Roggero and Dentrèves 2005, Aytekin et al. 2007, Francuski et al. 2009, Tüzün 2009) and in delimitation among the genera (Baracchi et al. 2011). However, this modern effective methodology has not been applied in the studies of cantharid beetles until now.

In Cantharidae, the venation of hind wings was suggested to be of diagnostic value in the subfamily level based on the comparative morphology by Brancucci (1980). But within the subfamily, the variables of the veins are shown to be quantitative in metric properties, which can not be studied well by the traditional morphometrics, so it remains unknown whether the hind wing morphology contributes to the delimitation of genera or species or not. Thus in the present study, we apply the landmark-based geometric morphometric method to quantify and analyze wing morphological features in nine species belonging to three genera of Cantharinae, including *Lycocerus* Gorham, 1889 (sensu Okushima 2005, more than 300 species in the world), *Prothemus* Champion, 1926 (60 species in total), and *Themus* Motschulsky, 1838 (approximately 250 species worldwide), which are all mostly distributed in the Oriental region. The central aim of the study is to evaluate wing shape variation and test the possible use of wing shape patterns for generic or specific taxonomy of Cantharinae.

## Material and method

### Sample collections

Hind wings of the following Cantharinae species (Table 1) are used in this study. Prior to geometric morphometric analysis, identification of specimens was performed using other morphological characters of adults (Yang 2010). The materials of the representative species are deposited in the Museum of Hebei University, Baoding, China (**MHBU**) and Institute of Zoology, Chinese Academy of Sciences, Beijing, China (**IZAS**) respectively. The left hind wing of each specimen (215 wings in total) was removed from the body and mounted in neutral balsam between a microscope slide and a cover slip. For each species, the chosen male and female specimens are subequal in number.

**Table 1.** The number of specimens of each species used in the GM analysis.

Specific name	Number of specimens	
	male	female
*Lycocerus asperipennis* (Fairmaire, 1891)	9	11
*Lycocerus metallescens* (Gorham, 1889)	12	15
*Lycocerus orientalis* (Gorham, 1889)	13	13
*Prothemus kiukiangensis* (Gorham, 1889)	10	11
*Prothemus limbolarius* (Fairmaire, 1900)	10	10
*Prothemus purpuripennis* (Gorham, 1889)	11	14
Themus (Telephorops) coelestis (Gorham, 1889)	14	18
Themus (Telephorops) impressipennis (Fairmaire, 1886)	10	12
Themus (Haplothemus) licenti Pic, 1938	12	10

### Data acquisition

**Figure 1.**
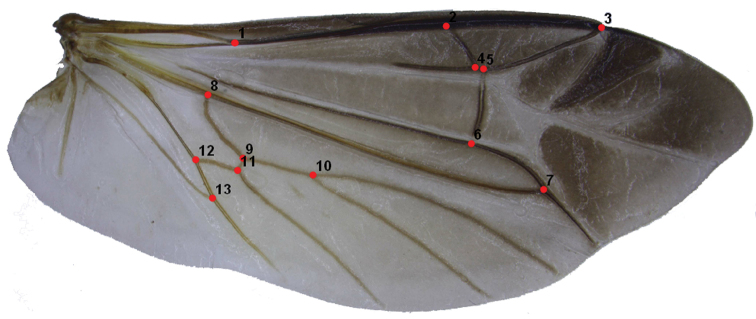
Hind wing of *Lycocerus asperipennis* showing digitizing landmarks.

The images of hind wings were captured using a stereomicroscope Nikon SMZ1500 and attached video camera Canon 450D connected to a HP computer. They were annotated using the TpsUtil software (Rohlf 2010a). The coordinates of the landmarks (13 landmarks in total, Table 2) were digitized by the TpsDig2.16 software (Rohlf 2010b) as shown in Fig. 1.

**Table 2.** Landmarks of hind wing (according to veins nomenclature system by Kukalová-Peck and Lawrence (1993).

No.	Junctions of veins	No.	Junctions of veins
1	ScP (Subcosta Posterior) and RA	8	MP_1+2_ and MP_3+4_
2	RA (Radius Anterior) and RA_3+4_	9	MP_3+4_ and CuA_1_ (Cubitus Anterior)
3	RA_1+2_ and RA_3+4_	10	MP_4_ and MP_3_
4	RA_3+4_ and r3 (radial crossvein)	11	CuA_1_ and CuA_2_
5	RA_3+4_ and r4	12	CuA and CuA_1+2_
6	r4 and RP (Radius Posterior)	13	AA (Anal Anterior) and CuA_3+4_
7	RP and MP_1+2_ (Media Posterior)		

### Geometric morphometric analyses

To examine the wing shape variation, the digitized landmark data is analyzed using MorphoJ software (Klingenberg 2011). The variability in the shape space is assessed using a Principal Component Analysis (PCA). To better visualize the shape variation, we present the average configuration of landmarks for each genus or species. Deformation grids are used to portray the resulting shape variations.

The relative similarity and discrimination of the genera or species is analyzed using Canonical Variates Analysis (CVA). CVA finds shape values that maximize group means relative to variation within groups, by assuming that covariate matrices are identical (Klingenberg 2010). This is an effective method for detecting differences among taxa. The statistical significance of pairwise differences in mean shapes is determined using permutation tests (10 000 replications) with Procrustes and Mahalanobis distances. Both tests are used to assess significance because *p*-values can differ due to the anisotropy (direction dependency) of shape variation (Klingenberg and Monteiro 2005).

To evaluate the role of wing size in discrimination among different genera or species, the centorid size (CS) was compared. In the absence of allometry, the CS is the only size measure uncorrelated with all the shape variables (Bookstein 1991). The CS values are compared for genera and species respectively, because as a measurement of overall size variation of wings, they are far more sensitive than conventional measurements (Klingenberg et al. 1998). One-way analysis of variance (ANOVA) and Tukey HSD pairwise comparisons are employed to determine significant differences among genera or species. For visualizing size differences among groups, a 95% confidence intervals of the mean is computed using SPSS 13.0 and plotted in EXCEL.

## Results

**Figure 2.**
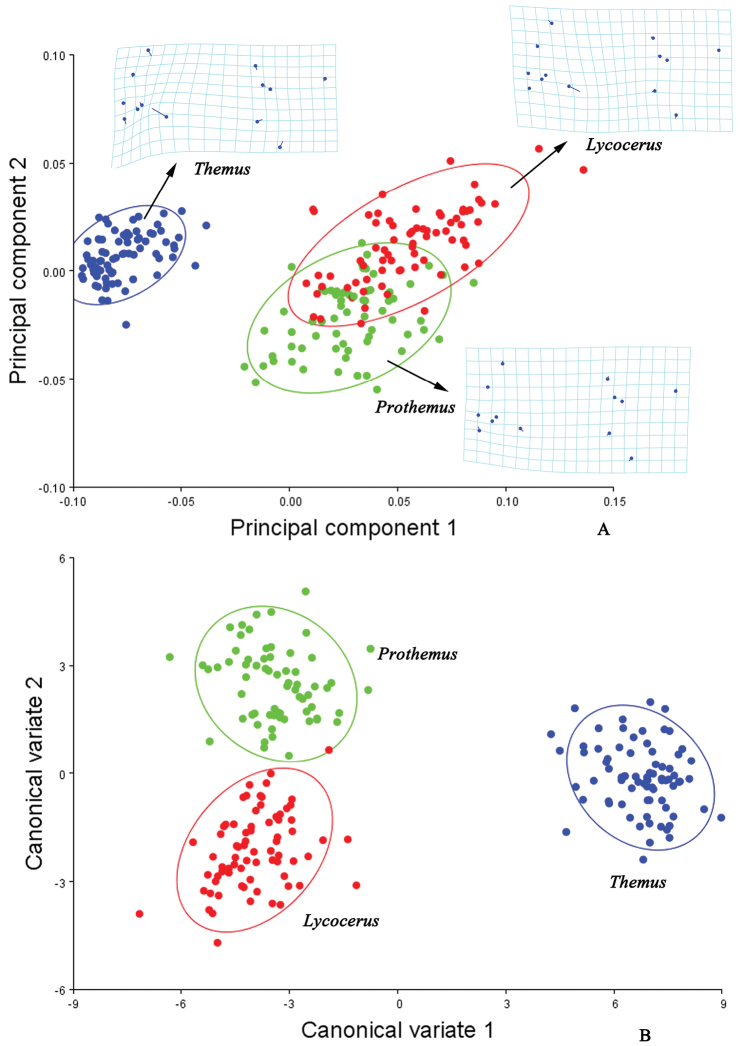
Shape variables of the hind wings in the genera of *Lycocerus*, *Prothemus* and *Themus*. **A** principal component analysis (PCA) of hind wing configuration. Plot of PC1 (74.39% of total variation) and PC2 (8.52% variation) showing 90% confidence ellipses of population means **B** canonical variate analysis (CVA) of same matrix, also showing 90% confidence ellipses of population means. The averaged shape of each genus is depicted as deformations using thin plate splines.

The shape variations of the hind wings in the genera *Lycocerus*, *Prothemus* and *Themus* is shown by the first two principal components of PCA (Fig. 2A). The thin plate spline visualizations show that the medial area (around by junctions Nos 9‒13) contributes most to the shape differences among the genera, especially the situation of the junction of MP_4_ and MP_3_ (No. 10) is most variable in *Themus*, while least in *Lycocerus*, and similar for the junction of ScP and RA (No. 1). Also, the junctions of r4 and RP (No. 6) and RP and MP_1+2_ (No. 7) appear more variable in *Themus* than in *Lycocerus* or *Prothemus*. Besides, the hind wing shape is more elongate in *Themus* than the other two genera. The centroid size (Fig. 6A, Table 7) is significantly different among the three genera (all *p*<0.05). The CVA scatterplot of shape differences for these genera (Fig. 2B) shows that each genus occupies different area. Mahalanobis distances among the three genera are significantly different in all pairwise comparisons (*p*<0.05), and Procrustes distances (*p*<0.05) are similar (Tables 3).

**Table 3.** Difference in the hind wing shapes among the genera *Lycocerus*, *Pothemus* and *Themus*. Mahalanobis distances (left) & Procrustes distances (right): *p*-values (above); distances between populations (below).

	*Lycocerus*	*Pothemus*	*Themus*	*Lycocerus*	*Pothemus*	*Themus*
*Lycocerus*	—	<.0001	<.0001	—	<.0001	<.0001
*Pothemus*	4.6396	—	<.0001	0.0456	—	<.0001
*Themus*	10.8932	10.446	—	0.1323	0.1088	—

**Figure 3.**
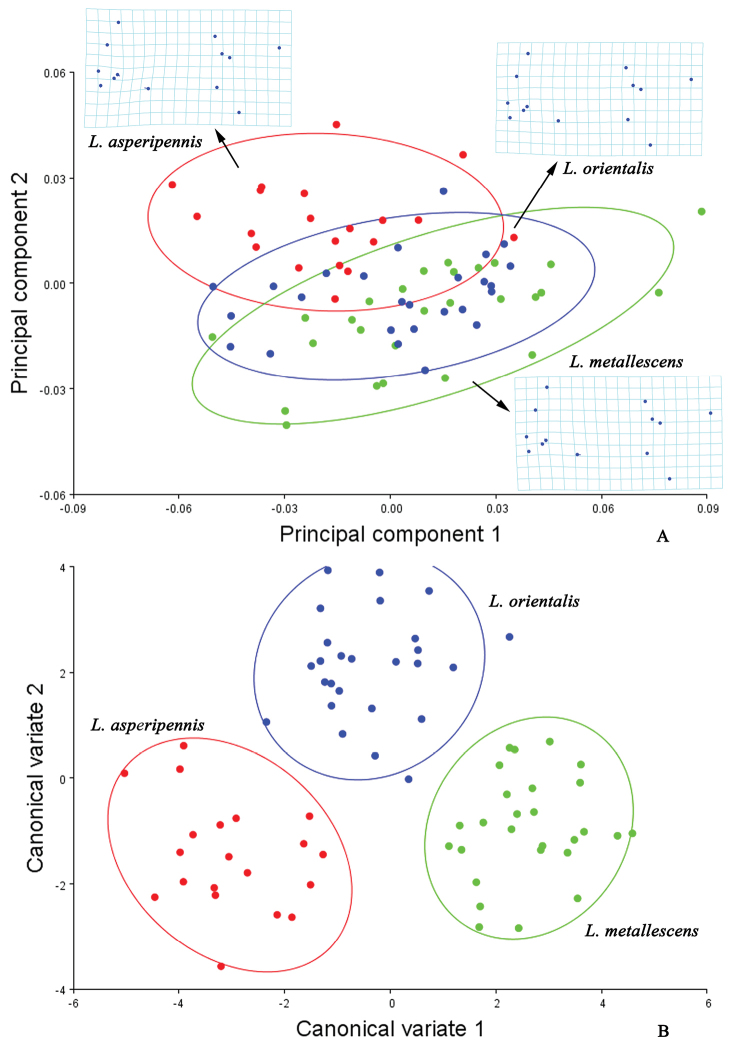
Shape variables of the hind wings in the *Lycocerus* species. **A** principal component analysis (PCA) of hind wing configuration. Plot of PC1 (49.02% of total variation) and PC2 (14.92% variation) showing 90% confidence ellipses of population means **B** canonical variate analysis (CVA) of same matrix, also showing 90% confidence ellipses of population means. The averaged shape of each species is depicted as deformations using thin plate splines.

**Figure 4.**
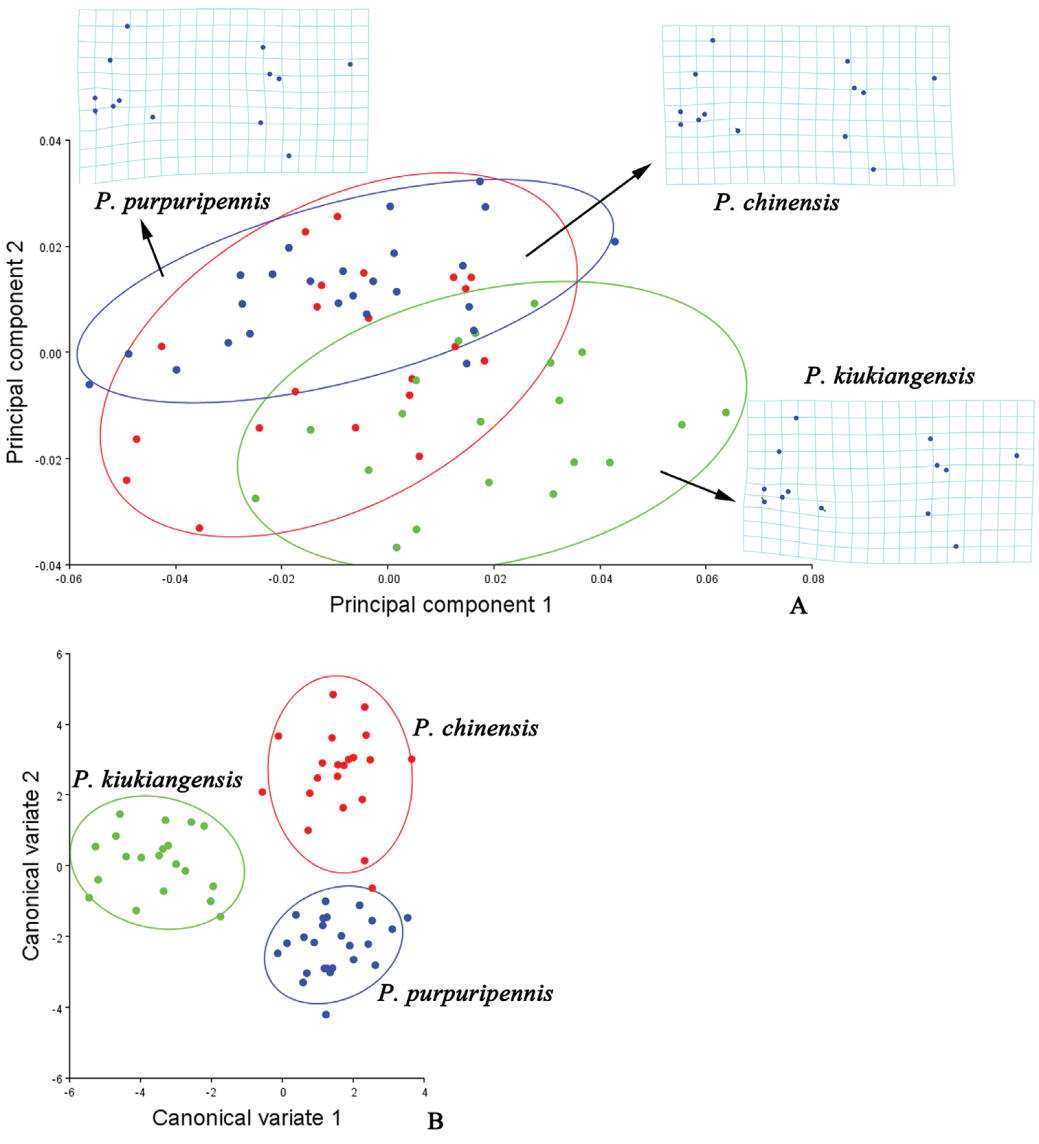
Shape variables of the hind wings in the *Prothemus* species. **A** principal component analysis (PCA) of hind wing configuration. Plot of PC1 (38.40% of total variation) and PC2 (15.88% variation) showing 90% confidence ellipses of population means **B** canonical variate analysis (CVA) of same matrix, also showing 90% confidence ellipses of population means. The averaged shape of each species is depicted as deformations using thin plate splines.

**Figure 5.**
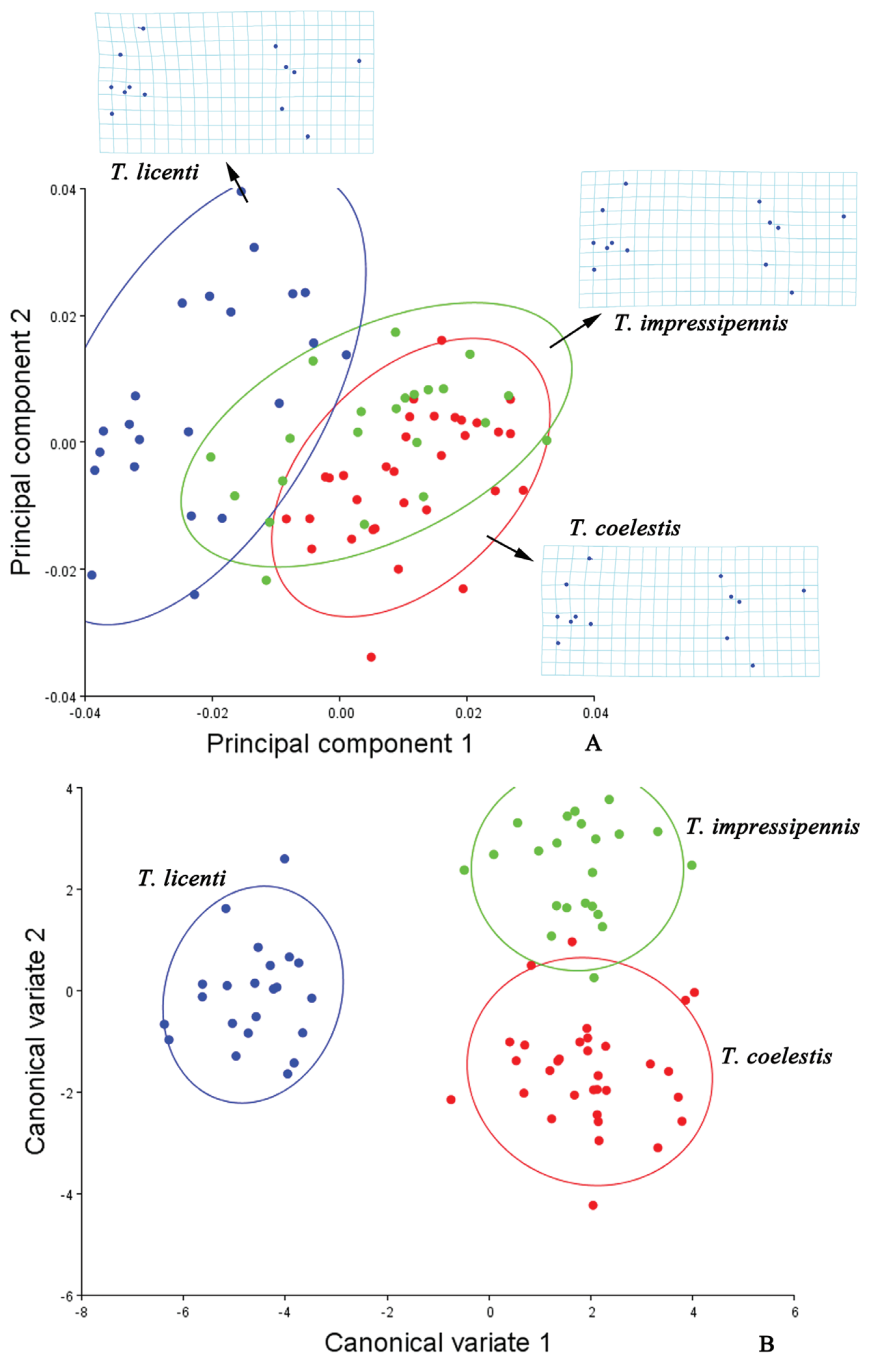
Shape variables of the hind wings in the *Themus* species. **A** principal component analysis (PCA) of hind wing configuration. Plot of PC1 (32.87% of total variation) and PC2 (16.48% variation) showing 90% confidence ellipses of population means **B** canonical variate analysis (CVA) of same matrix, also showing 90% confidence ellipses of population means. The averaged shape of each species is depicted as deformations using thin plate splines.

**Figure 6.**
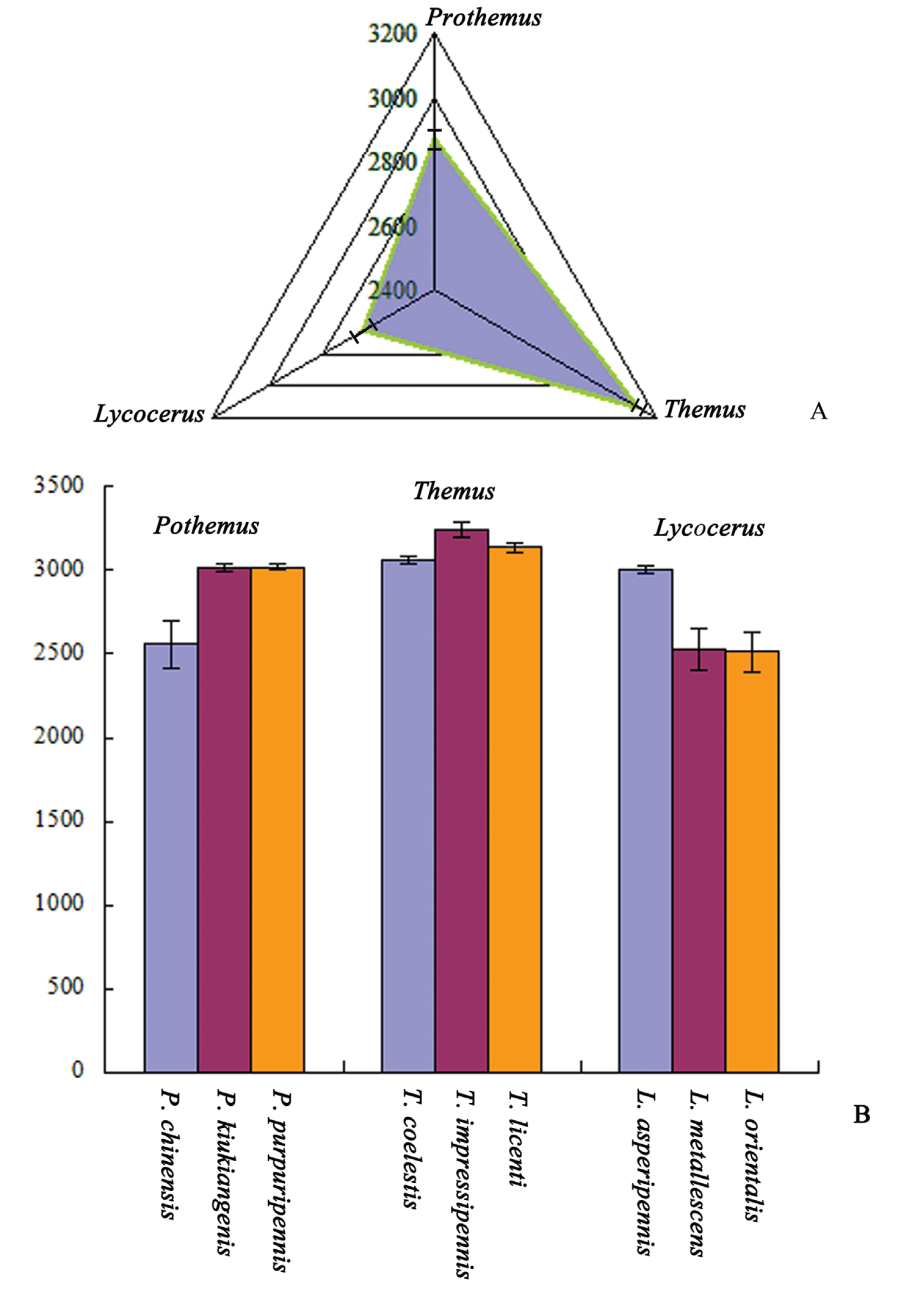
Comparisons of centroid size variables among different groups: **A** *Lycocerus*, *Prothemus* and *Themus* **B** *Lycocerus asperipennis*, *Lycocerus metallescens* and *Lycocerus orientalis*; *Prothemus chinensis*, *Prothemus kiukiangensis* and *Prothemus purpuripennis*; *Themus licenti*, *Themus coelestis* and *Themus impressipennis*.

In *Lycocerus* (Fig. 3A), the thin plate spline visualizations show that the junction of MP_4_ and MP_3_ (No. 10) is less variable in *Lycocerus orientalis* than in *Lycocerus asperipennis* or *Lycocerus metallescens*, and MP_3+4_ and CuA_1_ (No. 9) is more variable in *Lycocerus asperipennis* than the other two. In *Prothemus* (Fig. 4A), the junction of MP_4_ and MP_3_ (No. 10) is most variable in *Prothemus kiukiangensis*, while least in *Prothemus purpuripennis*, and AA and CuA_3+4_ (No. 13) is less variable in *Prothemus chinensis* than the other two. In *Themus* (Fig. 5A), the junction of ScP and RA (No. 1) is most variable in *Themus licenti*, while least in *Themus impressipennis*. The centroid size (Fig. 6B, Table 7) is significantly different between *Lycocerus asperipennis* and *Lycocerus orientalis* (*p*=0.001) or *Lycocerus metallescens* (*p*=0.001), *Prothemus chinensis* and *Prothemus kiukiangensis* (*p*=0.005) or *Prothemus purpuripennis* (*p*=0.002), but others are not (*p*>0.05). The CVA scatterplots of shape differences for each genus (Fig. 3B, 4B, 5B) all show that each species occupies different area. Mahalanobis distances among the three species of each genus are significantly different in all pairwise comparisons (*p*<0.05), and Procrustes distances are similar (*p*<0.05) (Tables 4, 5, 6).

**Table 4.** Difference in the hind wing shapes among the species of *Lycocerus*. Mahalanobis distances (left) & Procrustes distances (right): *p*-values (above); distances between populations (below).

	***Lycocerus metallescens***	***Lycocerus asperipennis***	***Lycocerus orientalis***	***Lycocerus metallescens***	***Lycocerus asperipennis***	***Lycocerus orientalis***
*Lycocerus metallescens*	—	<.0001	<.0001	—	<.0001	0.0466
*Lycocerus asperipennis*	5.6866	—	<.0001	0.0413	—	0.0003
*Lycocerus orientalis*	4.2970	4.4457	—	0.0182	0.0321	—

**Table 5.** Difference in the hind wing shapes among the species of *Prothemus*. Mahalanobis distances (left) & Procrustes distances (right): *p*-values (above); distances between populations (below).

	*Prothemus chinensis*	*Prothemus kiukiangensis*	*Prothemus purpuripennis*	*Prothemus chinensis*	*Prothemus kiukiangensis*	*Prothemus purpuripennis*
*Prothemus chinensis*	—	<.0001	<.0001	—	<.0001	0.0002
*Prothemus kiukiangensis*	5.7352	—	<.0001	0.0376	—	<.0001
*Prothemus purpuripennis*	4.8174	5.5146	—	0.0247	0.0381	—

**Table 6.** Difference in the hind wing shapes among the species of *Themus*. Mahalanobis distances (left) & Procrustes distances (right): *p*-values (above); distances between populations (below).

	*Themus licenti*	*Themus coelestis*	*Themus impressipennis*	*Themus licenti*	*Themus coelestis*	*Themus impressipennis*
*Themus licenti*	—	<.0001	<.0001	—	<.0001	<.0001
*Themus coelestis*	6.7942	—	<.0001	0.0363	—	0.0001
*Themus impressipennis*	6.8548	3.9959	—	0.0311	0.016	—

## Discussion

The result of PCA shows that the shape differences of the hind wings among the genera *Lycocerus*, *Prothemus* and *Themus* (Fig. 2A) are mostly associated with the junctions of MP_4_ and MP_3_ (No. 10), ScP and RA (No. 1), r4 and RP (No. 6) and RP and MP_1+2_ (No. 7), and the shape of *Themus* is much more different from that of *Lycocerus* than *Prothemus*. And those variations within each genus (Figs 3A, 4A, 5A) appear in one or two junctions, which are either same to that of the genera or not, such as MP_3+4_ and CuA_1_ (No. 9) in *Lycocerus* and AA and CuA_3+4_ (No. 13) in *Pothemus*. This demonstrates that the shape differences among genera are much more variable than that within genus, and the variations among the species of each genus are different from one another.

The CVA results (Figs 2B, 3B, 4B, 5B) show that the three genera and the species of each genus are all successfully discriminated, since that Mahalanobis and Procrustes distances (Tables 3–6) for each group are significantly different (*p*<0.05). It suggests that the hind wing shape is useful for discrimination of both genus and species in Cantharinae by the geometric morphometrics. Also, the hind wing size is considered to be valuable in delineating the genera, but its role is uncertain for the species because of the inconsistent results in the three genera (Table 7).

**Table 7.** Tukey HSD for the CS among different groups: *p*-values (above); mean differences (below). Asterisk (*) indicates the mean difference is significant at the 0.05 level.

CS among different genera			
	***Lycocerus***	***Prothemus***	***Themus***
*Lycocerus*	—	0.006	0
*Prothemus*	218.52316401(*)	—	0.001
*Themus*	483.54109456(*)	-265.01793055(*)	—
**CS among the species of *Lycocerus***			
	***Lycocerus asperipennis***	***Lycocerus metallescens***	***Lycocerus orientails***
*Lycocerus asperipennis*	—	0.001	0.001
*Lycocerus metallescens*	474.67493257(*)	—	1
*Lycocerus orientails*	489.29359311(*)	14.61866054	—
**CS among the species of *Prothemus***			
	***Prothemus chinensis***	***Prothemus kiukiangensis***	***Prothemus purpuripennis***
*Prothemus chinensis*	—	0.005	0.002
*Prothemus kiukiangensis*	-456.74308033(*)	—	1
*Prothemus purpuripennis*	-460.37428735(*)	-3.63E+00	—
**CS among the species of *Themus***			
	***Themus coelestis***	***Themus impressipennis***	***Themus licenti***
*Themus coelestis*	—	0.711	0.998
*Themus impressipennis*	-183.8607895	—	0.992
*Themus licenti*	-79.25669086	104.6040987	—

Herein it can be concluded that the hind wing shape is useful for the discriminations of genera and species of Cantharinae. The geometric morphometrics represents a reliable tool not only in the taxonomic research but also in further study on the evolution of the hind wing shape of cantharid beetles.
